# Make and Break Your Own Hand: A Review of Hand Anatomy and Common Injuries

**DOI:** 10.21980/J8PH0Z

**Published:** 2020-01-15

**Authors:** Gabriel Sudario, Alisa Wray, Robin Janson

**Affiliations:** * University of California, Irvine, Department of Emergency Medicine, Orange, CA; ^ Indiana University—Purdue University at Indianapolis, Department of Occupational Therapy, Indianapolis, IN

## Abstract

**Audience:**

The target audience for this small group session is emergency medicine residents, primarily for use in didactic conference. This session can also be utilized with medical students, or faculty looking to review relevant hand anatomy and common injuries.

**Introduction:**

Three-dimensional (3D) printing is an emerging technology that has the ability to produce highly accurate anatomic, cellular and medical device models. Limited research has shown promise in teaching anatomy,^1^ congenital heart disease^2^ and surgical pre-operative planning.^3^ Despite this potential, there is sparse evidence of 3D printing emergency medicine residency education. The Model of Clinical Practice of Emergency Medicine specifies content for American Board of Emergency Medicine certification and requires proficiency in a wide breadth of medical topics including upper extremity and hand injuries.^4^ The concepts of hand anatomy and function rely heavily on understanding spatial relationships between bones, tendons and ligaments. The instructional strategy of working with 3D printed hand models aligns with these learning goals. This project seeks to directly incorporate 3D printing into the orthopedic curriculum of emergency medicine residents during a required weekly didactic educational session.

**Educational Objectives:**

By the end of this session, learners should be able to name and identify all bones of the hand; arrange and construct an anatomically correct bony model of the hand; build functional phalangeal flexor and extensor tendon complexes onto a bony hand model; describe the mechanism of injury, exam findings, and management of the tendon injuries Jersey finger, Mallet finger, and central slip rupture; draw/recreate injury patterns on a bony hand model; and describe the mechanism of injury, exam findings, imaging findings, and management of scapholunate dissociation, perilunate dislocation and lunate dislocation, Bennett’s fracture, Rolando fracture, Boxer’s fracture and scaphoid.

**Educational Methods:**

This session was delivered in a small group session which utilized educational methods grounded in constructivist learning such as complex problem-solving, social negotiation, and spatial learning.

**Research Methods:**

Verbal feedback was obtained after the session.

**Results:**

Overall learners found the session engaging, interactive, and especially useful in demonstrating relevant hand anatomy and injuries. Learners felt that hands-on experience with the hand models reinforced knowledge and helped them better identify injuries in a spatial fashion.

**Topics:**

Extremity bony trauma, dislocations/subluxations, tendon injuries.

## USER GUIDE

[Table t1-jetem-5-1-sg1]

**Table t1-jetem-5-1-sg1:** 

List of Resources:	
Abstract	1
User Guide	3
Learner Materials	7
Instructor Materials	12

**Learner Audience:**

Medical Students, Interns, Junior Residents, Senior Residents

**Time Required for Implementation:**

Instructors will need nearly ten hours of preparation for this session. We recommend allowing eight hours for 3D printing the hand models along with two hours for reviewing pre-reading along with organizing supplies for the session. Although the printing of the hand models takes approximately eight hours, the instructor doesn’t have to be present the entire time because most 3D printers are “set it and forget it” and print on their own.

**Recommended Number of Learners per Instructor:**

4–6 but will vary on the number of models that are printed.

**Topics:**

Orthopedics, traumatic bony injuries, hand injuries, tendon injuries, anatomy.

**Objectives:**

By the end of this session, learners will be able to:

1. Name and identify all bones of the hand.
2. Arrange and construct an anatomically correct bony model of the hand.
3. Demonstrate the physical exam steps to assess for normal motor, nerve, and tendon function of the hand.
4. Build functional phalangeal flexor and extensor tendon complexes onto the bony hand model.
5. Describe the mechanism of injury, exam findings, and management of the following tendon injuries: Jersey finger, Mallet finger, and central slip rupture.
6. Arrange a bony hand model into the following common carpal dislocations: scapholunate dissociation, perilunate dislocation, and lunate dislocation.
7. Describe the mechanism of injury, exam findings, imaging findings and management of the following injuries: scapholunate dissociation, perilunate dislocation, and lunate dislocation.
8. Draw the following fracture patterns onto a bony hand model: Bennett’s fracture, Rolando fracture, Boxer’s fracture, and scaphoid fracture.
9. Describe the mechanism of injury, exam findings, imaging findings, and management of the following injuries: Bennett’s fracture, Rolando fracture, Boxer’s fracture, and scaphoid fracture.

### Linked objectives and methods

In developing our objectives and methods, our needs assessment comes from the Model of the Clinical Practice of Emergency Medicine from the American Board of Emergency Medicine.^4^ Our small group session includes the core content of extremity bony trauma with focus on dislocations/subluxations, fractures, sprains, and strains. This instructional intervention rests mostly on constructivist learning theory which states that “knowledge is constructed by learners as they attempt to make sense of experiences.”^7^ Our small group places emphasis on ideal conditions for instruction in the constructivist model, which includes having a complex learning goal, social negotiation, and ownership over learning. By building a functional model of the hand and then using it to identify relevant injury patterns, learners are able to conceptualize these injuries by building knowledge through understanding anatomy and the pathology that occurs when this anatomy is disrupted. Leveraging the importance of spatial learning, this instructional strategy of working with 3D printed models aligned well with our session objectives.

### Recommended pre-reading for facilitator

- Bowen WT, Slaven E. Evidence-based management of acute hand injuries in the emergency department (Trauma CME). *Emerg Med Pract*. 2014;16(12):1–25.
- Janson R. 3D printable hand bone anatomy model. https://www.myminifactory.com/object/3d-print-hand-bone-anatomy-model-81620. Accessed April 4, 2019.

### Learner responsible content (LRC)

- Bowen WT, Slaven E. Evidence-based management of acute hand injuries in the emergency department (Trauma CME). *Emerg Med Pract*. 2014;16(12):1–25.

### Approximate cost of items to create this innovation

$30 (please note this is cost not including a 3D printer)

- $20 - Cost of plastic spool for printing four hands
- $5 - Elastic cords
- $5 - “Tendon” ribbon

### Associated Materials

- Hand Bone Anatomy Model by Janson
- HB R Metacarpals Horizontal.stl
- HB R Metacarpals Vertical.stl
- HBM R Carpus.stl
- HBM R Phalanges.stl

### Preparation

*3D Printing Preparation***:** Please note that this section is an adaptation (with permission) of the small group session “Hand Bone Anatomy Model” by Dr. Robin Janson and all images are copyright Dr. Robin Janson.^6^

- *Equipment Needed:* Computer and 3D printer.
- *3D Model Files:* https://iu.box.com/v/3D-HandOT.
- *Prerequisite Skills:* Printer Control/Slicer Software.
- *Materials:* PLA filament, white round elastic co 1/16″ (1.5mm) diameter, 3.3 yards (220cm) in length. May use any string <1.5mm in diameter. Blue ribbon 3/8 inch in width cut into 5cm lengths. Red ribbon 1/8 inch in width cut into 5 cm lengths, adhesive tape.
- *Tools:* Pliers, sanding knife, craft knife, 1.5mm rod.

#### 3D Printing the Hand Bone Anatomical Model

1. Download the following digital files to your computer: HBM R Carpus, HBM R Metacarpals (vertical or horizontal), HBM R Phalanges.
2. In deciding vertical versus horizontal orientation, vertical orientation is preferred because it produces an overall better bone appearance (in contrast to horizontal prints), and requires less material and less post-processing time (support/raft removal). If able, deselect support from printing into the bone holes. If unsuccessful printing vertically, try additional support material at the bone bases or print horizontally. 3D printing the metacarpals lying flat on the build plate increases stability. Unfortunately, this orientation increases print surface contact with supports and will require time post-processing to remove supports. When preparing prints in this orientation, deselect support from the bone holes (if able). Otherwise, removal of support material from the metacarpal bone holes can be very challenging and may require drilling as a last resort!
3. Open Printer Control/Slicer software, import and prepare files for 3D printing. We recommend the following print settings:
  - Layer Height: 0.2 mm
  - Shells: 3
  - Infill: 10%
  - Supports: yes
  - Rafts: yes
4. 3D print the prepared bone files. The following are estimated times and material needed for each file:
  - Carpus: 1.25 hours, 5.7 meters
  - Metacarpals Vertical: 3 hours, 9.5 meters
  - Metacarpals Horizontal: 2.75 hours, 11 meters
  - Phalanges: 2.75 hours, 9.6 meters
  Total estimated print time: 7–8 hours
  Total estimated materials: 26 meters of filament[Fig f2-jetem-5-1-sg1]
5. Post processing: While employing safe practices (eye protection, hand safety, etc.) carefully remove rafts and supports using pliers and craft knife. Smooth all rough surfaces using a sanding file. Remove any support material from bone holes by carefully pushing a sturdy 1.5mm metal rod (eg, a large paper clip) through holes.[Fig f1-jetem-5-1-sg1]

#### Other Material Preparation

Prepare the elastic cord by cutting six-30cm length strands and two-20cm length strands. The cords have been color coded in the following instructions as a visual aid for this activity. If desired, each cord may be color-coded using markers. Also, if needed, carpal bone holes may be color-coded to match corresponding color of cord.

Cut blue ribbon into two 5cm length sections. With one blue ribbon section, cut a vertical 1cm line into the center of the ribbon. In the other blue ribbon, cut one end of the ribbon 2.5cm, vertically to create two “tails” at the end of the ribbon. Cut red ribbon into two 5cm sections.

### Results and tips for successful implementation

This session is best implemented when instructors have adequately prepped learning materials for the session. In terms of the 3D printing, the above session is a detailed tutorial on how to approach 3D printing the model and can be easily followed by those with minimal experience with the technology. If there is access to a maker’s space or technologist who is facile with the technology, we recommend utilizing these resources for the 3D printing process.

In terms of pre-session preparation, we recommend that instructors attempt construction of the hand model prior to the session. This not only provides insight into the learner process but can identify rate limiting steps that can be expedited in the session. For example, in our session, learners were having trouble threading elastic cord through the bone models due to small pieces of material in the threading holes. Had we attempted to build these hands ahead of time, we could have cleared the openings and prevented this frustrating step.

We also recommend having all material organized and labeled prior to the session. As discussed below, colored elastic cords were immensely helpful in orienting our learners in how to articulate the model. We pre-cut and then colored these items with permanent marker prior to the session. An additional step that could have led to more efficient model building would be to color the model bone elastic cord holes with the same color that matched the color of elastic cords being used. We also pre-cut ribbon that was used to model tendons, and packed each complete model in a separate bag for learners. Each bag contained the bone models, elastic cord, ribbon, tape, a permanent marker, and a threading tool.

We implemented this session on a group of 24 emergency medicine residents and medical students rotating on our emergency medicine clerkship. Verbal feedback was obtained after the session. Overall learners found the session engaging, interactive and especially useful in demonstrating relevant hand anatomy and images. Time was a significant limitation in our session. We had the session planned for 90 minutes, but most teams were unable to complete all aspects of the session. This meant that some teams were unable to even begin the injury matrix exercise at the end of the session. The most rate limiting step was the hand articulation portion, where learners had difficulty finding the correct orifices that corresponded to the various elastic cords. As discussed above, color coordinated cords and markings on the bone model could decrease the time needed building the model.

Pre-building models could have been a feasible alternative to improve the timing problems with this session. Learners could be given disarticulated bones to arrange in anatomical order, but then skip the building step and be given the pre-built model to have adequate time to explore tendon function/injuries and bony dislocations and fractures.

Verbal feedback indicated that building hand tendon models was useful for constructing knowledge in tendon function and injuries in learners, but the rudimentary nature of these models made it difficult to have the tendons “function” as hoped. When residents would pull on the tendon complex created, often times the model would just bend the finger in a non-anatomic way. This could be mitigated by bracing the hand model and allowing only the finger to move. Despite not being able to fully recreate anatomical movement with these models, they were still helpful in having learners visualize how flexor/extensor tendons and hand bones interact in a spatial manner.

Finally, the injury matrix allowed learners to synthesize information from pre-reading and the hand building session. The matrix does take significant time to complete, and we did not have a group complete this component of the session. We recommend taking advantage of the collaborative nature of Google Docs and assigning specific rows of the matrix to specific teams. This would allow learners to dive more deeply into 1–2 specific topics and provides a complete matrix that could be used by all learners for future reference.

### Pearls

Learning pearls are nicely summarized in the Hand Injury Matrix that learners complete as part of this activity. Answers to this session are located in this link: https://docs.google.com/document/d/1WQ4Px2wdLf6IiitfIMnyMK1jDyjTTKuT-yEItIYLZeI/edit?usp=sharing

## Supplementary Information



## Figures and Tables

**Table t2-jetem-5-1-sg1:** Hand Injury Matrix

Injury	Activity	Mechanism of Injury	Exam Findings	Imaging Findings	Management
**TENDON INJURIES**					
**Jersey Finger**	Model this injury on your hand model/tendon model.				
**Mallet Finger**					
**Central Slip Ruptures**					
**DISSOCIATIONS**					
**Scapholunate Dissociation**	Recreate this dislocation on your bony hand model.				
**Perilunate Dislocation**					
**Lunate Dislocation**					
**FRACTURES**					
**Bennett’s Fracture**	With sharpie, draw this fracture pattern on your bony hand model.				
**Rolando Fracture**					
**Boxer’s Fracture**					
**Scaphoid Fracture**					

**Table:** Hand Injury Matrix Answers

Injury	Activity	Mechanism and Injury Description	Exam Findings	Imaging Findings	Management
**TENDON INJURIES**					
**Jersey Finger**	Draw visual depiction of this tendon injury.	Forced extension of the DIP joint during active flexion. Closed injury of the FDP tendon distal to DIP joint.	DIP swelling, volar DIP tenderness, impaired DIP flexion.	XR without bony abnormalities. Ultrasound may have some use in differentiating full thickness rupture.	Dorsal hand and wrist splint with intrinsic plus position. Referral to hand surgery in 7 days.
**Mallet Finger**		Forced flexion of the DIP joint during extension. Injury of the extensor tendon distal to the DIP joint.	Digit held in flexion.	XR may show avulsion fracture.	Splint and immobilization of the DIP in extension. PIP joint should have full ROM. Hand surgery referral.
**Central Slip Injury**		Usually laceration over PIP joint with blunt trauma or dislocation with tendon complex disruption.	Unable to extend DIP with resistance. Abnormal Elson’s or Modified Elson’s test. Boutonniere’s deformity chronically.	XR without bony abnormality. May use to look for foriegn body.	Splint in extension with ortho referral in 7 days.
**DISSOCIATIONS**					
**Scapholunate Dissociation**	Recreate this dislocation on your bony hand model.	High impact FOOSH with wrist hyperextension and ulnar deviation.	Wrist swelling, tenderness over scapholunate joint, decreased ROM.	Scapholunate diastasis > 3mm.	Thumb spica splint. Referral to hand surgery.
**Perilunate Dislocation**		High impact FOOSH with wrist hyperextension.	Wrist swelling, deformity of wrist with tenderness over the dorsal scapholunate joint, decreased ROM.	Displacement of the capitate with retention of the lunate articulation with the radius. “Jumbled Carpus”	Emergent hand consult
**Lunate Dislocation**		High impact FOOSH with wrist hyperextension.	Wrist swelling, deformity of wrist with tenderness over the dorsal scapholunate joint, decreased ROM.	Displacement and rotation of the lunate. “Spilled teacup”.	Emergent hand consult
**FRACTURES**					
**Bennet’s Fracture**	With sharpie, draw this fracture pattern on your bony hand model.	Axial loading	Tenderness at base of thumb.	2-part intra-articular fracture dislocation or subluxation of the base of the thumb metacarpal.	Reduction, thumb spica and urgent hand referral.
**Rolando Fracture**		Axial loading	Tenderness at base of thumb.	Y-shaped comminuted fracture dislocation of the base of the thumb metacarpal	Reduction, thumb spica and urgent hand referral.
**Boxer’s Fracture**		Usually axial loading during punching/fight. Fracture of the fifth metacarpal neck with volar displacement of the metacarpal head.	Tenderness over 5th metacarpal.	Metacarpal neck fracture with possible angulation. Severe fractures occur if angulation is greater than 45° or if rotation is greater than 20° external reduction is required.	Closed reduction. Ulnar gutter splint or dorsal slab.
**Scaphoid Fracture**		FOOSH.	Snuffbox tenderness, tenderness with axial loading of the thumb.	Scaphoid view. Normal in 20% of cases.	Thumb spica and repeat xray in 14 days for those you are suspicious of.

**Figure 1 f1-jetem-5-1-sg1:**
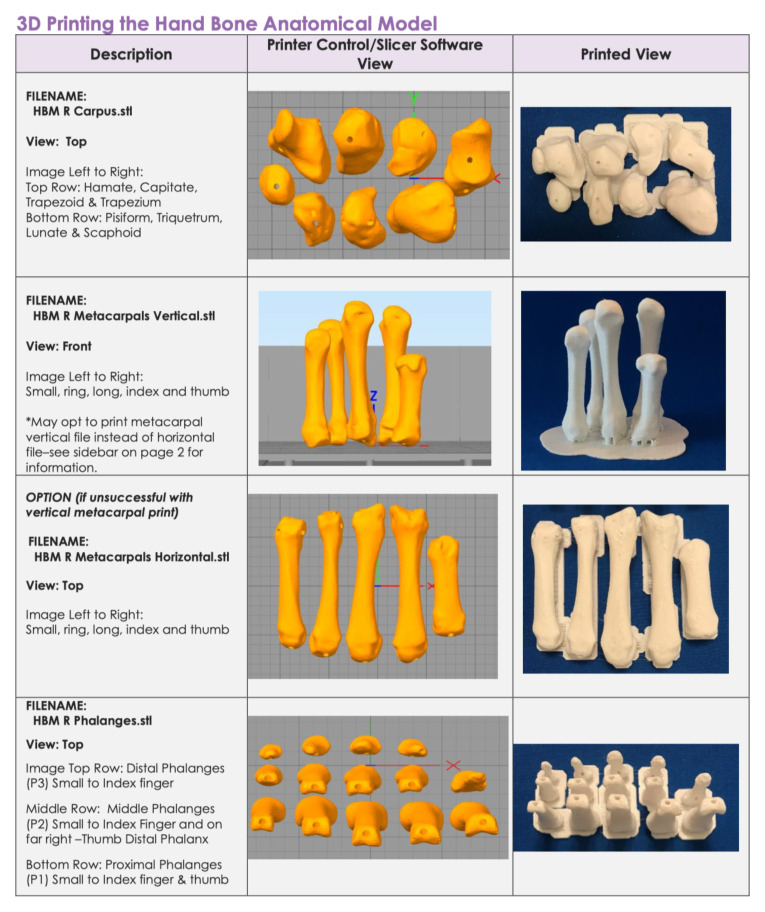
File and Printing Information

**Figure 2 f2-jetem-5-1-sg1:**
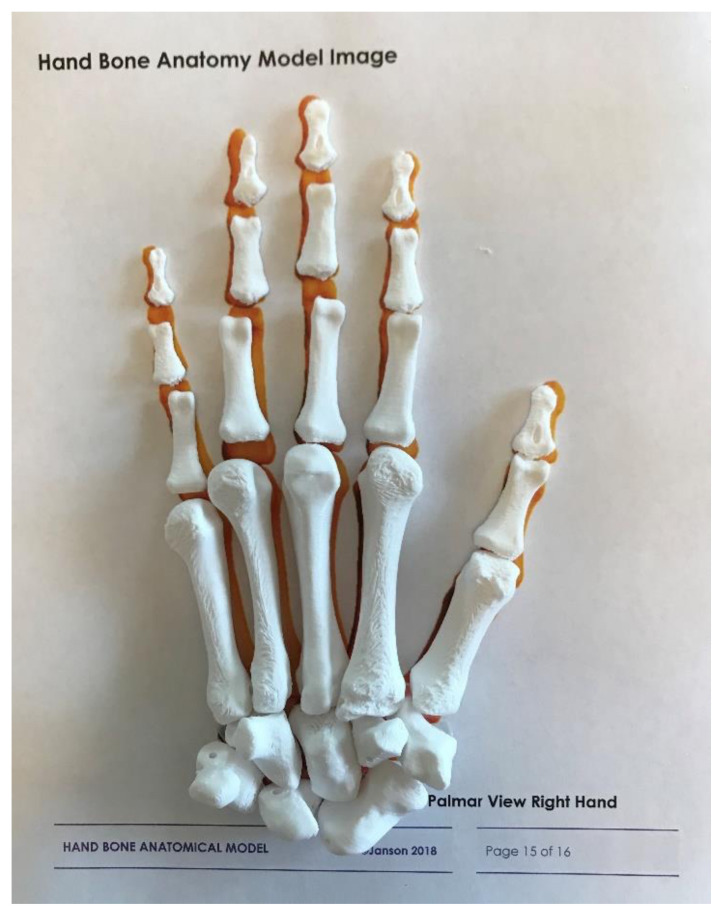
Hand bones in anatomical configuration on reference page.

**Figure 3:**
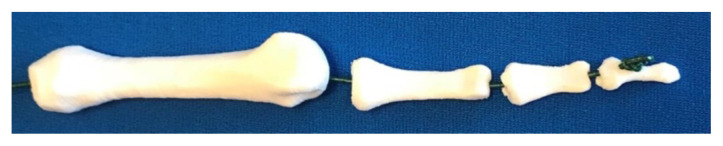
Threaded Finger Bones

**Figure 4:**
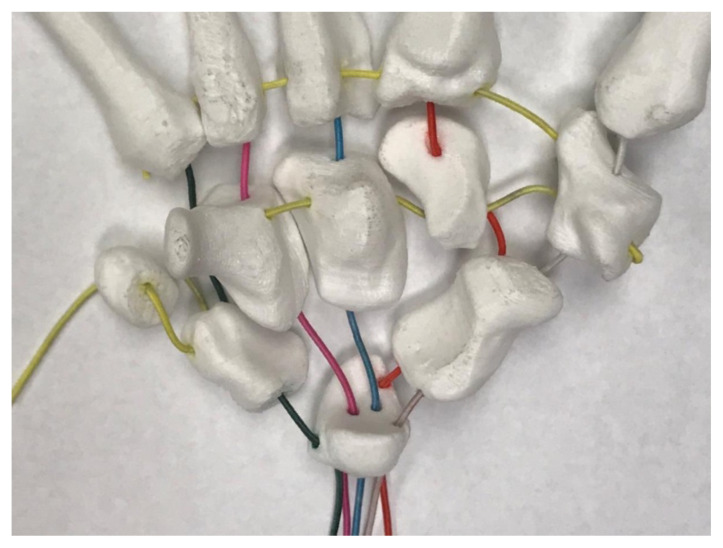
Color-Coded Threading Pattern of the Carpus (Palm Up Volar View)

**Figure 5:**
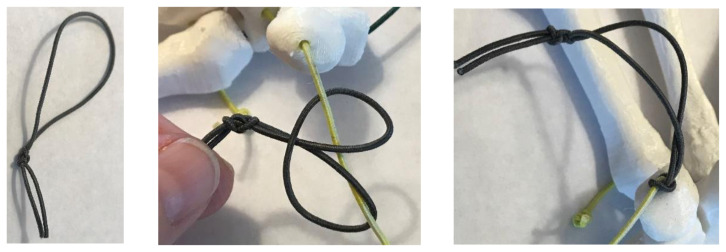
Create tension loop and apply as shown to the cord exiting the pisiform and the cords exiting the lunate.

## LEARNER MATERIALS

### Make and Break Your Own Hand: A Review of Hand Anatomy and Common Injuries

#### Small Group Application Exercise (sGAE)

##### Learner responsible content (LRC)

Bowen WT, Slaven E. Evidence-based management of acute hand injuries in the emergency department (Trauma CME). *Emerg Med Pract*. 2014;16(12):1–25.

##### Small group application exercise (sGAE)

Please note that sections 1–3 are an adaptation (with permission) of the small group session “Hand Bone Anatomy Model” by Dr. Robin Janson, and all images are copyright Dr. Robin Janson.^6^

1. The Pre-Quiz:
  - Arrange bones in correct anatomical order (palm/volar orientation) on a blank sheet of paper. Note that due to the shape of the wrist bones, the wrist bones will not lay in proper anatomical position.
  - Identify and label the three bone groups of the hand.
  - Identify and name each individual wrist bone.
  - Give the names of the rows of the wrist bones. List which wrist bones are in each respective row.
  - Identify and label special features (bony prominences) of the rest of the hands.
  - Identify and label the joints of the fingers, thumb, and wrist.
2. The Disarticulated Hand
  - Using the Hand Bone Anatomy Model Image on page 15 of the supplemental pdf “Hand Bone Anatomy Model by Janson”, match the individual hand bones (palm up) to their anatomical location on the diagram (as shown below). Reference Hand Bone Anatomy Diagrams on pages 11–13 of the supplemental pdf.
  - Identify and label:
    - bone groups: phalanges, metacarpals, and carpals
    - individual carpal bones
3. Articulate the hand:
  - Articulate the hand bones by threading the bones with the cord (40cm length for each digit) from a distal (fingertip) to proximal (towards the wrist) direction. Follow the directions below while referencing Figures 3, 4, and 5 below for threading instructions.
    - First, tie a large knot at the end of each cord before threading (knots must be larger than the 2.0mm distal phalangeal bone holes). This knot will anchor the cord within the distal phalanx.
    - Thumb (white): distal phalanx→ proximal phalanx→ metacarpal→ trapezium (articular surface) → scaphoid (palmar hole) → lunate (palmar hole).
    - Index (red): distal phalanx→ middle phalanx→ proximal phalanx→ metacarpal→ trapezoid (articular surface) → scaphoid (dorsal holes) → lunate (dorsal holes).
    - Long (blue): distal phalanx→ middle phalanx→ proximal phalanx→ metacarpal→ capitate (articular surface) → lunate (middle hole).
    - Ring (pink): distal phalanx→ middle phalanx→ proximal phalanx→ metacarpal→ hamate (radial side articular surface) → lunate (hole in middle of concave articular surface).
    - Small (green): distal phalanx→ middle phalanx→ proximal phalanx→ metacarpal→ hamate (ulnar side articular surface) → triquetrum (hole through length of bone) → lunate (hole at top of V portion).
    - 6th String (yellow): Thread through the metacarpal bases of the small finger (starting with knotted end on the ulnar side) → ring finger→ long finger→ index finger→ (skip thumb) → trapezium (loop through bone) → trapezoid → capitate→ hamate→ triquetrum→ pisiform.
    - After threading the hand bones, slide bones distally to cinch into place. Apply removable tension loops to secure the bones in place, one over the cord exiting the pisiform and the other over the group of five cords exiting the lunate. Do not trim cord ends short if the model will be dissembled and reassembled.
4. Build Finger Tendons
  - Build a Flexor Tendon
    - Find the blue ribbon with a middle slit; this represents the flexor digitorum superficialis (FDS).
    - Find the thinner, red ribbon; this represents the flexor digitorum profundus (FDP).
    - Using tape, the two ribbons and any additional online resource, create a rudimentary but functioning flexor tendon complex.
  - Describe the functional limitation of a patient who sustains a proximal FDP injury.
  - Describe the functional limitation of a patient who sustains a proximal FDS injury.
  - What physical exam maneuvers can differentiate between an FTP and FPS injury?
  - Build an extensor tendon
    - Find the blue ribbon split into two; this represents the extensors lateral bands.
    - Find the thinner red ribbon; this represents a central slip.
    - Using tape, the two ribbons and any additional online resource, create a rudimentary but functioning extensor tendon complex.
  - Describe the functional limitation of a patient who sustains a central slip rupture.
  - What is the name of the chronic deformity that can occur with a central slip rupture?
  - What physical exam maneuver can reveal a central slip injury?
5. Complete the Hand Injury Matrix either electronically or with the below matrix.
  - Electronically: This is a Google Doc, linked here: https://docs.google.com/document/d/1WQ4Px2wdLf6IiitfIMnyMK1jDyjTTKuT-yEItIYLZeI/edit?usp=sharing. Please note that this document is read only. To make an editable copy, simply click “File”→“Make a Copy.”

[Table t2-jetem-5-1-sg1]

## INSTRUCTOR MATERIALS

### Make and Break Your Own Hand: A Review of Hand Anatomy and Common Injuries

#### Small Group Application Exercise Answer Key (sGAE Answer Key)

1. The Pre-Quiz: Answers to these questions will be covered by completing the full exercise. See page 11 of the supplemental pdf “Hand Bone Anatomy Model by Janson”, for full anatomy reference.
2. The Disarticulated Hand: Learners will place bones onto page 15 of the supplemental pdf “Hand Bone Anatomy Model by Janson”, and reference page 11 of the supplemental pdf “Hand Bone Anatomy Model by Janson”, to complete this anatomy identification section.
3. Hand Bone Articulation: Learners will use Figures 3, 4, and 5 above (or images on page 8 and 9 of the supplemental pdf “Hand Bone Anatomy Model by Janson”), to complete this section by building the full, articulated hand model. See page 7 of the supplemental pdf “Hand Bone Anatomy Model by Janson”, for a photo of a completed hand model.
4. Build Finger Tendons and Analyze Injury Patterns:
  - Build a Flexor Tendon: The 2^nd^ through 5^th^ digits flexor apparatus consists of the flexor digitorum profundus (FDP) and the flexor digitorum superficialis (FDS). The FDS is superficial to the FDP and inserts on the volar aspect of the middle phalanx. The FDP inserts at the volar aspect of the distal phalanx. 5
  - Describe the functional limitation of a patient who sustains a proximal FDP injury.
    - Injury to the proximal FDP impairs distal interphalangeal (DIP) joint flexion while maintaining proximal interphalangeal (PIP) joint flexion.^8^
  - Describe the functional limitation of a patient who sustains a proximal FDS injury.
    - Injury to the FDS tendon would limit PIP joint flexion while DIP flexion would remain intact.^8^
  - What physical exam maneuvers can differentiate between an FDP and FDS injury?
    - To assess function of the FDP tendon, examiners should test for DIP flexion with the PIP held in extension. To assess for function of the FDS, all other fingers should be held in extension while finger being assessed is tested for PIP flexion. Because four of the FDP tendons are linked, holding all other fingers in extension prevents FDP flexion which could cause misinterpreted flexion on exam. 9
  - Build an extensor tendon.
    - Extensor complex involves an extensor expansion that divides into a central clip that attaches to the middle phalanx. It then forms two lateral bands that attach to the base of each distal phalanx.^10^
  - Describe the functional limitation of a patient who sustains a central slip rupture.
    - Central slip rupture would cause decreased extension of the PIP joint against resistance. Extension may be preserved when the lateral bands remain intact. 10
  - What is the name of the chronic deformity that can occur with a central slip rupture?
    - The Boutonniere deformity is a delayed complication after injury with central slip disruption. This results in unopposed FDS motion displacing the lateral bands volar. The deformity is seen as hyperextension of the DIP joint with lateral bands acting as flexors, flexing the PIP joint. 11
  - What physical exam maneuver can reveal a central slip injury?
    - The modified Elson’s test has the patient place the dorsal middle phalanges of the injured finger and matching contralateral finger in opposition with PIPs flexed at 90 degrees. They are asked to push the fingers together and extend the DIP joint. Central slip injury is revealed by having more extension at the DIP joint due to unopposed extension of the lateral bands without a functional central slip extending the PIP joint. 12
5. Hand Injury Matrix Answers available either electronically or with the below written answers and matrix.
  - Electronically: This is a Google Doc, linked here: https://docs.google.com/document/d/1nWlMGi1QnPSb8Y40xAs-NlzfheEN00AE_zdd3oTYvsM/edit?usp=sharing

##### Jersey Finger

This injury is caused by forced extension of the DIP joint during active flexion. This causes closed injury of the FDP tendon distal to DIP joint. On exam, one would expect DIP swelling, volar DIP tenderness and impaired DIP flexion. XR will usually show no bony abnormalities, but ultrasound may be useful in diagnosing full thickness rupture and/or bony avulsions. All patients are considered surgical candidates and should be placed in a dorsal hand and wrist splint with intrinsic plus position with referral to hand surgery in 7 days. 11,13

##### Mallet Finger

This injury is caused by forced flexion of the DIP joint during extension causing disruption of the extensor tendon or bony avulsion injury distal to the DIP joint. On exam, patients will have distal, dorsal pain with inability to actively extend the DIP joint. XRs could identify avulsion fracture but can also be normal. Finger should be splinted in extension with full range of motion (ROM) of the PIP joint maintained. 11

##### Central Slip Injury

This injury usually occurs as a PIP joint dislocation with tendon disruption. It can also present as a laceration over the PIP joint. Exam usually presents with PIP joint deformity and some patients may have acute Boutonniere deformity (flexion of the PIP joint and hyperextension of the DIP joint). The DIP joint will have no active extension. Radiographs may show dislocation but are usually normal. The PIP joint is splinted in extension with DIP and metacarpophalangeal (MCP) joint remaining mobile. Hand referral should be obtained within 7 days. 11

##### Scapholunate Dissociation

Scapholunate dissociation results from a high-impact fall on outstretched hand (FOOSH). These injuries are seen where there is hyperextension and ulnar deviation of the wrist. It represents the earliest stages of progressive perilunar instability.^14^ On exam, patient will have a swollen, painful wrist. On radiographs, one should evaluate for scapholunate diastasis greater than 3mm in width. Patients should be splinted in a thumb spica splint and referred urgently to hand surgery. 5

##### Perilunate and Lunate Dislocation

These two injuries are discussed together and represent even further progression of perilunar instability. They are both caused by high-impact FOOSH and will also present with wrist swelling and tenderness. On lateral radiograph, perilunate dislocation will show dorsal displacement of the capitate with retained lunate/radius articulation. In contrast, lunate dislocations will show displacement and rotation of the lunate which is call the “spilled tea-cup” sign. Both injuries require emergent hand surgery consult. 15

##### Bennett’s and Rolando Fractures

Bennett’s and Rolando fractures are both intra-articular fractures of the base of the thumb metacarpal base, usually cause by axial loading injuries.^5^ Bennett’s fractures are a two-part fracture dislocations of the base of the metacarpal whereas Rolando fractures are comminuted, 3-part, Y-shaped, comminuted fracture dislocations of base of the thumb metacarpal. These fractures present with tenderness and swelling at the base of the thumb, and radiographs usually reveal the above fracture findings. Both injuries require reduction, thumb spica splint placement, and urgent referral to a hand surgeon.^16^

##### Boxer’s Fracture

The Boxer’s fracture is a fracture of the fifth metacarpal neck. They are usually caused by striking a solid object with a clenched fist. Patients will be tender over the 5^th^ MCP and possibly have deformity in that area. Radiographs will reveal metacarpal neck fracture. Reduction is accomplished by flexing the MCP, PIP and DIP of the affected hand to 90 degrees and applying axial traction with volar force onto the metacarpal.^17^ An ulnar gutter splint should be applied with the MCP joints in at least 70 degrees of flexion to prevent collateral ligament shortening.^16^ Patient should be discharged with a hand surgery referral.

##### Scaphoid Fracture

The scaphoid fracture is commonly associated with a FOOSH injury. Missed scaphoid injury can lead to significant morbidity given risk of avascular necrosis and scapholunate advanced collapse.^5^ Classically, patients will have anatomic snuffbox tenderness, but clamp sign and resisted supination pain have been shown to have higher positive predictive value.^18^ Plain films should include PA, lateral, oblique and scaphoid views of the wrist, with up to 20% of fractures not demonstrated on radiographs.^19^ Application of a thumb spica splint is recommended in patients with radiographic confirmed fracture or in those with high clinical suspicion for the injury. These patients require referral to hand surgery. Those with high suspicion injuries but negative radiographs will require repeat radiographs in 10–14 days.^5^
