# Supplementary material for: Make and Break Your Own Hand: A Review of Hand Anatomy and Common Injuries
Source: J Educ Teach Emerg Med. 2020 Jan 15;5(1):SG1–SG16. doi: 10.21980/J8PH0Z (PMC10332531; doi:10.21980/J8PH0Z)
Supplement: Supplementary file 1 [file jetem-5-1-sg1-supp1.pdf]

# Hand Bone Anatomy Model

**By Robin Janson, OTD, MS, OTR, CHT**

### About

The 3D Printable Hand Bone Anatomy Model was created to provide learners* with a low-cost anatomical model. It consists of 27 disarticulated bones with holes for articulation.

Files for 3D printing the model along with this manual may be downloaded from https://iu.box.com/v/3D-HandOT.

After 3D printing the bones complete the Pre-Quiz before engaging in the learning activities. After completing the learning activities, complete the Post-Quiz and compare pre- and post-quiz scores to assess learning gains.

### Model Development

Digital hand bones from Upper Limb Bones (Left) by the author, HandOT, (https://www.thingiverse.com/thing:1352085) were scaled to a larger size and mirrored to create a right hand in Simplify3D. An articulated human hand bone skeleton prepared by the Charles H. Ward Company was referenced for carpal bone hole placements. Original bone files from **Human Hand Bones –Thumb** by Siderits (Thingiverse #15342).

**This manual is intended for university/college level students; however, may be adapted for younger learners.*

### Table of Contents


| Table of Contents | Page |
|---|---|
| 3D Printing Summary | 2-3 |
| Hand Bone Pre-Quiz | 4 |
| Learning Activities | |
| I. Disarticulated Hand Bone Introduction | 5 |
| II. Disarticulated Hand Bone Mastery | 6 |
| III. Articulated Hand Bones | 7-9 |
| IV. Hand Joints | 10 |
| References | 10 |
| Hand Bone Anatomy Forms | 11-14 |
| Hand Bone Post-Quiz | 15 |


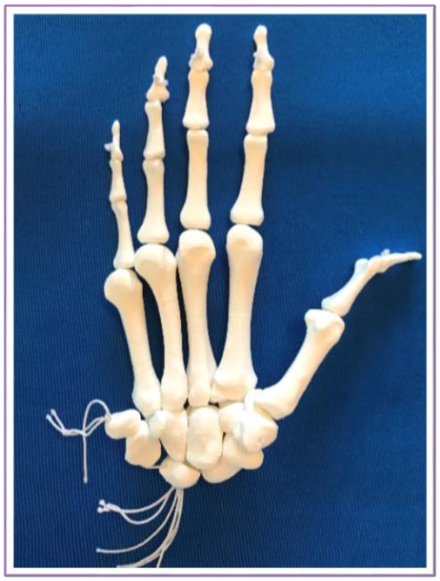

**Hand Bone Anatomy Model Articulated-Right**

## Equipment

- Computer
- 3D Printer (FFF)

## Prerequisite Skills

- Printer Control/Slicer Software
- 3D Printer Operation

## Materials

- PLA Filament
- White Round Elastic Cord 1/16" *(1.5mm)* diameter & 3.3 yards *(220cm)* length. May use any string ≤ 1.5mm diameter.

## Tools

- Pliers
- Sanding file
- Craft knife
- 1.5mm diameter rod

 Copyright © 2018 Robin Janson

# 3D Printing the Hand Bone Anatomical Model

1. **Download** the digital files to your computer.

   - HBM R Carpus
   - HBM R Metacarpals *(vertical or horizontal– see sidebar for information)*
   - HBM R Phalanges

2. **Open Printer Control/Slicer software, import, and prepare files for 3D printing.** (see page 3 for file information)
   *Estimated time: 15 minutes*

   Recommended Print Settings

   | | |
   |---|---|
   | Layer Height | .2mm |
   | Shells | 3 |
   | Infill | 10% |
   | Supports | Yes |
   | Rafts | Yes |

   **Export prepared/sliced files**

3. **3D Print Prepared Bone Files**

   Estimated Print Times & Material Used

   | | | |
   |---|---|---|
   | Carpus | 1. 75 hours | 5.7 meters |
   | Metacarpals | | |
   | Vertical Print | 3 hours | 9.5 meters |
   | *Or-Horizontal Print* | *2.75 hours* | *11 meters* |
   | Phalanges | 2.75 hours | 9.6 meters |

   *Total Estimated Print Time: 7-8 hours*
   *Total Estimated Material: 26 meters*
   *Estimated Material Cost $2.60 (filament cost @ $0.10/m)*

4. **Post-Processing of Printed Bones** *Estimated time: 15 minutes with metacarpals printed vertically and 45 minutes if printed horizontally*
   While employing safe practices (eye protection, hand safety, etc.) carefully remove rafts and supports using pliers and craft knife. Smooth all rough surfaces using a sanding file. Remove any support material from bone holes by carefully pushing a sturdy 1.5mm metal rod (e.g. a large paper clip) through holes.

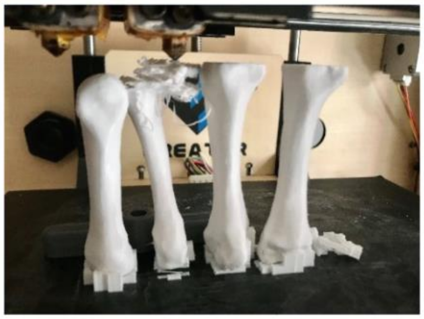

## Metacarpal Bones Print Vertically or Horizontally?

**Vertical Orientation (Preferred)** produces an overall better bone appearance (in contrast to horizontal prints), requires less material and less post-processing time (support/raft removal). If able, deselect support from printing into the bone holes. If unsuccessful printing vertically, try additional support material at the bone bases or print horizontally. *Photo above shows ring finger tipping resulting in a print fail.*

**Horizontal Orientation**

3D printing the metacarpals lying flat on the build plate increases stability. Unfortunately, this orientation increases print surface contact with supports and will require more time post-processing to remove the supports. When preparing prints in this orientation, deselect support from the bone holes (if able). Otherwise, removal of support material from the metacarpal bone holes can be very challenging (and may require drilling as a last resort!).

 **Copyright © 2018 Robin Janson**

# 3D Printing the Hand Bone Anatomical Model

| Description | Printer Control/Slicer Software View | Printed View |
|---|---|---|
| **FILENAME:**<br>**HBM R Carpus.stl**<br><br>**View: Top**<br><br>Image Left to Right:<br>Top Row: Hamate, Capitate, Trapezoid & Trapezium<br>Bottom Row: Pisiform, Triquetrum, Lunate & Scaphoid | 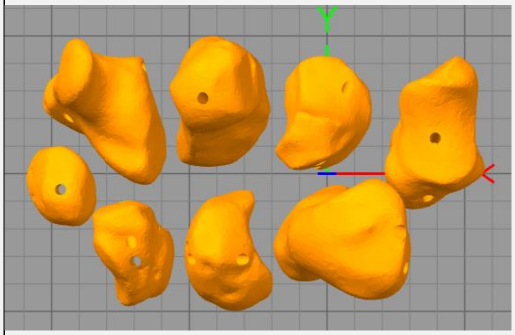 | 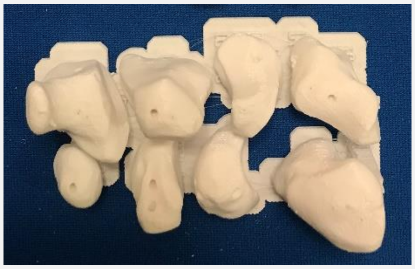 |
| **FILENAME:**<br>**HBM R Metacarpals Vertical.stl**<br><br>**View: Front**<br><br>Image Left to Right:<br>Small, ring, long, index and thumb<br><br>*May opt to print metacarpal vertical file instead of horizontal file–see sidebar on page 2 for information. | 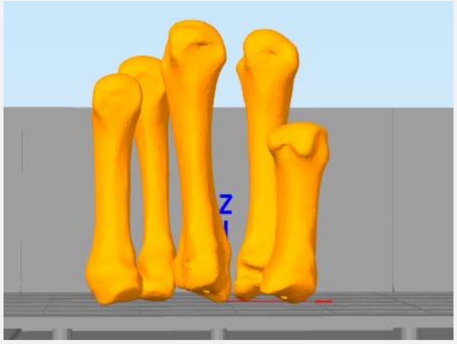 | 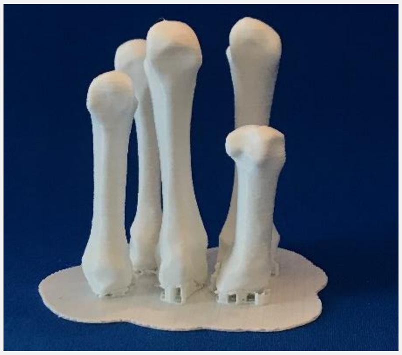 |
| ***OPTION (if unsuccessful with vertical metacarpal print)***<br><br>**FILENAME:**<br>**HBM R Metacarpals Horizontal.stl**<br><br>**View: Top**<br><br>Image Left to Right:<br>Small, ring, long, index and thumb | 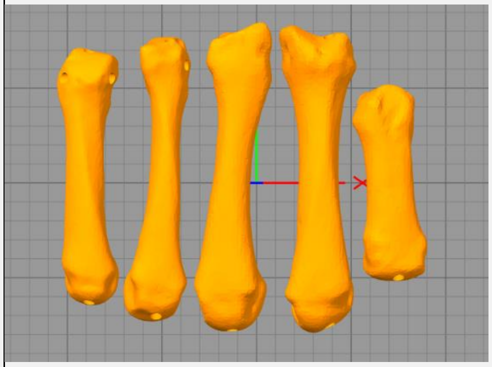 | 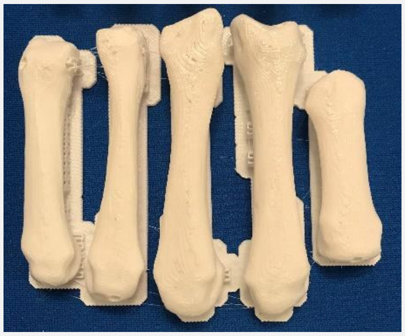 |
| **FILENAME:**<br>**HBM R Phalanges.stl**<br><br>**View: Top**<br><br>Image Top Row: Distal Phalanges (P3) Small to Index finger<br><br>Middle Row: Middle Phalanges (P2) Small to Index Finger and on far right –Thumb Distal Phalanx<br><br>Bottom Row: Proximal Phalanges (P1) Small to Index finger & thumb | 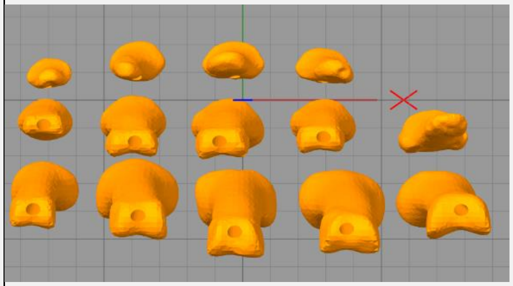 | 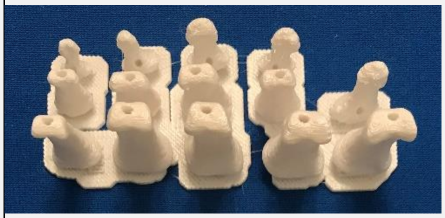 |

 Copyright © 2018 Robin Janson

## Pre-Quiz

**Instructions: 3D print the hand bones before completing this quiz. Complete this pre-quiz prior to engaging in the learning activities to assess your current knowledge of hand bone anatomy and identify areas to focus learning.**

1. Arrange bones in correct anatomical order (palm up/volar orientation) on a blank sheet of paper. Note that due to the shape of the wrist bones, the wrist bones will not lay in proper anatomical position.
2. Identify and label the three bone groups of the hand.
3. Identify and name each individual wrist bone.
4. Give the names of the rows of the wrist bones. List which wrist bones are in each respective row.
5. Identify and label as many special features (bony prominences) of the wrist bones as you can.
6. Identify and label the joints of the fingers, thumb and wrist.
7. Identify the three parts of the long bones of the hand?

**Answers are provided in the upside box below.**

**Score and save your pre-quiz for later comparison to the post quiz.**

**ANSWERS (Max Points = 61)**

1. Reference Hand Bone Model Diagram on page 5 for correct placement of bones. One point per correctly placed bone. Max Points= 27
2. Phalanges, metacarpals, and carpals. One point per correctly identified group. Max Points = 3
3. Scaphoid, lunate, triquetrum, pisiform, trapezium, trapezoid, capitate, hamate (pp. 5 & 12). One point per bone. Max Points=8
4. Proximal Row: scaphoid, lunate, triquetrum, pisiform / Distal Row: trapezium, trapezoid, capitate, hamate (pp. 5 & 12). One point per bone. Max Points=10 (distal row, proximal row, and the 8 bones)
5. Hamate – hook of hamate, trapezium – tubercle, scaphoid- tubercle, distal pole, waist, & proximal pole (p. 12). One point per feature. Max Points=6
6. Finger: DIP, PIP, MP & CMC; Thumb: IP, MP, CMC; intercarpal (between proximal and distal carpal rows) (p. 6). One point per joint group. Max Points=7

 Copyright © 2018 Robin Janson

## Learning Activities

### I. Disarticulated Hand Bone Anatomy – BASIC INTRODUCTION

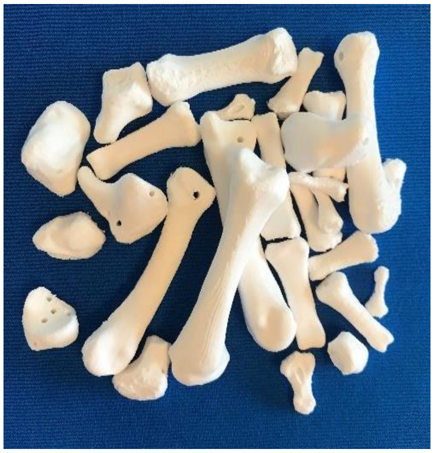

**Learning Objectives:**

- Identify the three bone groups of the hand.
- Identify the three parts of a long bone.
- Place disarticulated hand bone groups in approximate anatomical position.

1. Using the **Hand Bone Anatomy Model Image** on page 14, match the individual hand bones (palm up) to their anatomical location on the diagram (as shown below). Reference Hand Bone Anatomy Diagrams on pages 11-13 for bone locations and names.
2. Identify and label bone groups: phalanges, metacarpals and carpals.
3. Identify and label the three parts of the phalanges and metacarpals: head, shaft, and base.

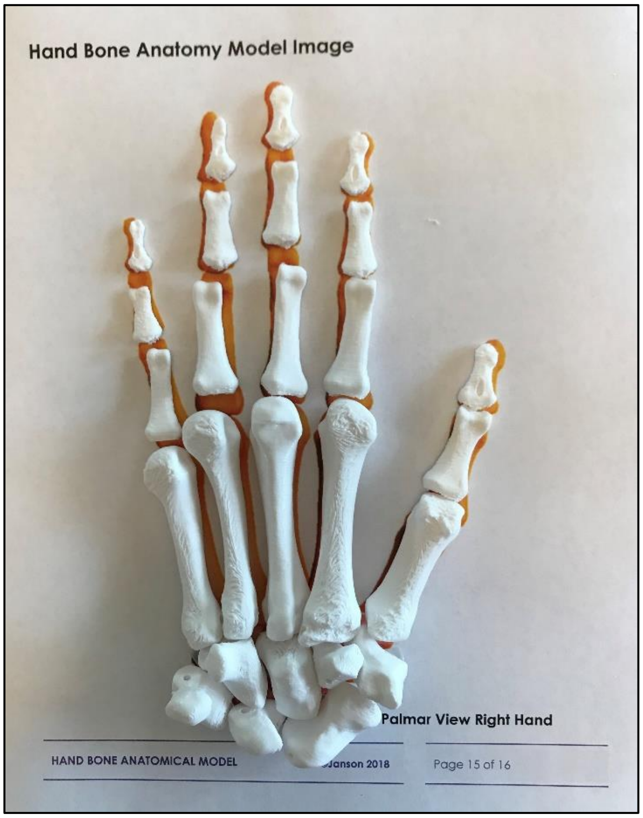


**Distal Phalanx**

**Middle Phalanx**

**Proximal Phalanx**

**Metacarpal**

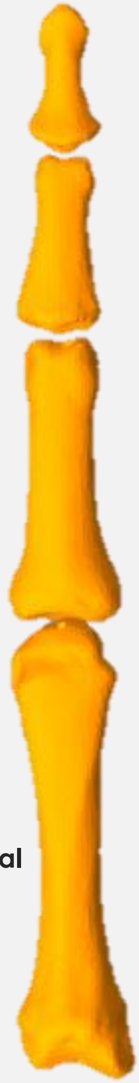

 **Copyright © 2018 Robin Janson**

## Learning Activities

### II. Disarticulated Hand Bone Anatomy - MASTERY

**Learning Objectives:**

- Name all 27 bones of the hand.
- Identify special features (bony prominences) of select carpal bones.
- Place disarticulated hand bones in correct anatomical position.

1. Arrange bones in correct anatomical order (palm up orientation) on a blank sheet of paper. May use 2-sided tape to help hold bones in place (especially carpal bones).
2. Identify and label:
    a) bone groups:  phalanges, metacarpals, and carpals
    b) individual carpal bones
    c) long bone features: (phalanges and metacarpals): head, shaft, and base
    d) carpal bone features: trapezium tubercle, hook of hamate, proximal and distal poles of the scaphoid, scaphoid waist, and scaphoid tubercle
3. Reference the Hand Bone Anatomy Model Diagrams on pages 11-13 or an anatomy text to check accuracy.

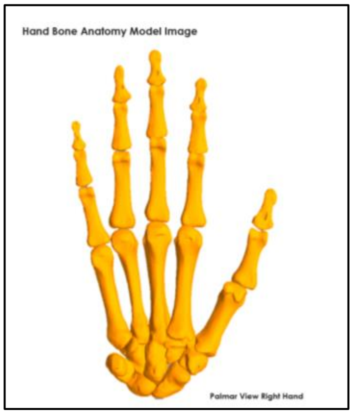


### Phalanges (14)

Distal  (P3)      = 5
Middle  (P2)    = 4
Proximal (P1)  = 5
*Note: thumb has two phalanges (proximal & distal)*

### Metacarpals (5)

### Carpals (8)

**Proximal Row:** Scaphoid, Lunate, Triquetrum, Pisiform
**Distal Row:** Trapezium, Trapezoid, Capitate, Hamate

### Memory Aids

Carpal Bone Mnemonic:
*"**S**teve **L**eft **T**he **P**arty To **T**ake **C**athy **H**ome"*
Steve = Scaphoid
Left = Lunate
The = Triquetrum
Party = Pisiform
To = Trapezium
Take  = Trapezoid
Cathy = Capitate
Home = Hamate

Thumb (metacarpal) swings (articulates) on a trapezium.

Think of the capitate and its central location within the carpus as the "capitol" of the carpus.

 Copyright © 2018 Robin Janson

## Learning Activities

### III. Hand Bone Articulation

**Learning Objectives:**

- Name all 27 bones of the hand.
- Identify special features (bony prominences) of select carpal bones.
- Assemble an articulated hand bone anatomical model.
- Name the joints of the hand.

1. Prepare the elastic cord by cutting six- 30cm length strands and two- 20cm length strands. The cords have been color coded in the following instructions as a visual aid for this activity. *If desired, each cord may be color-coded using markers. Also, if needed, carpal bone holes may be color-coded to match corresponding color of cord.*

**Color Coding**

| **Digit** | **Color** |
|---|---|
| Thumb | White *(uncolored)* |
| Index | Red |
| Long | Blue |
| Ring | Pink |
| Small | Green |
| **Additional Strings** | |
| $6^{th}$ String | Yellow |
| Tension Cords | Black |

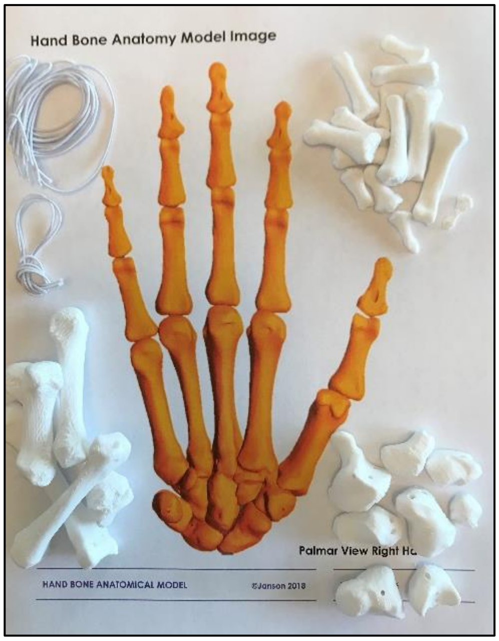


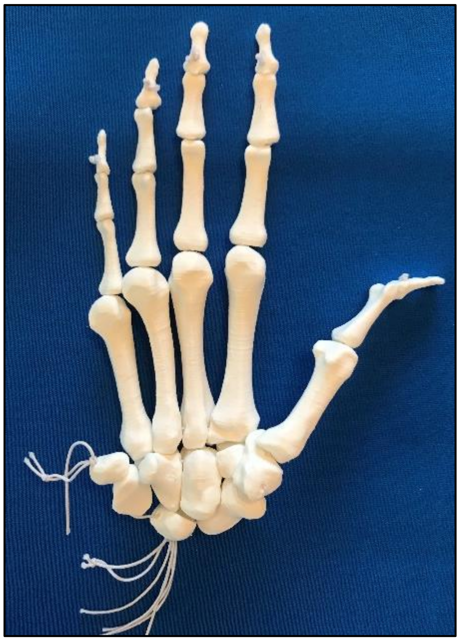

 Copyright © 2018 Robin Janson 

## Learning Activities

### III. Hand Bone Articulation -continued

**2. Arrange hand bones** on the Hand Bone Anatomy Model Image as shown on page 5.

**3. Articulate the hand bones,** by threading the bones with the cord (40cm length for each digit) from a distal (fingertip) to proximal (towards the wrist) direction. Follow the directions below while referencing the image on page 8 showing the color-coded threading pattern:

1. **First,** tie a **large knot** at the end of each cord before threading (knots must be larger than the 2.0mm distal phalangeal bone holes). This knot will anchor the cord within the distal phalanx.
2. **Thumb (white)**: distal phalanx→ proximal phalanx→ metacarpal→ trapezium (articular surface) → scaphoid (palmar hole) → lunate (palmar hole)
3. **Index (red):** distal phalanx→ middle phalanx→ proximal phalanx→ metacarpal→ trapezoid (articular surface) → scaphoid (dorsal holes) → lunate (dorsal holes)
4. **Long (blue)**: distal phalanx→ middle phalanx→ proximal phalanx→ metacarpal→ capitate (articular surface) → lunate (middle hole)
5. **Ring (pink)**: distal phalanx→ middle phalanx→ proximal phalanx→ metacarpal→ hamate (radial side articular surface) → lunate (hole in middle of concave articular surface)
6. **Small (green)**: distal phalanx→ middle phalanx→ proximal phalanx→ metacarpal→ hamate (ulnar side articular surface) → triquetrum (hole through length of bone) → lunate (hole at top of V portion).
7. **6th String (yellow)**: Thread through the metacarpal bases of the small finger (starting with knotted end on the ulnar side)→ ring finger→ long finger→ index finger→(skip thumb)→ trapezium (loop through bone)→ trapezoid → capitate→ hamate→ triquetrum→ pisiform

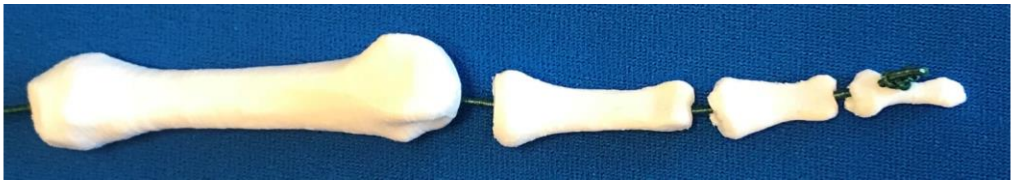

 Copyright © 2018 Robin Janson

## III. Hand Bone Articulation -continued

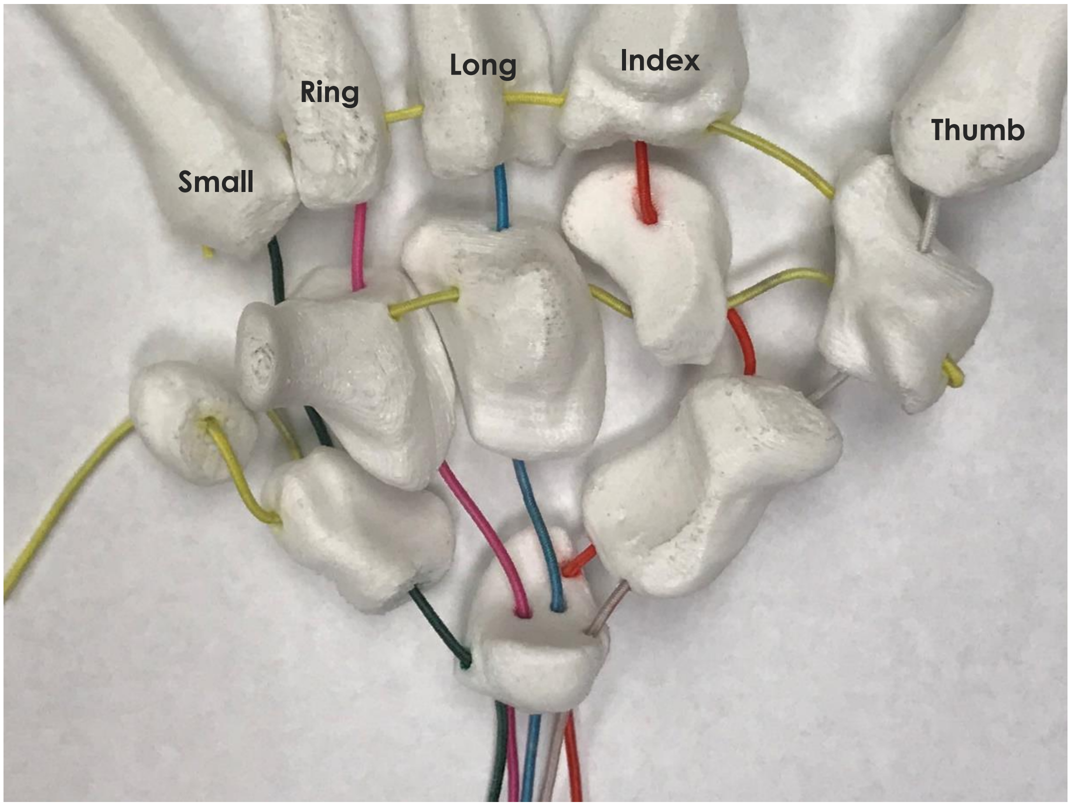


**Color-Coded Threading Pattern for the Carpus (Palm Up-Volar View)**

8. **After threading the hand bones,** slide bones distally to cinch into place. Apply removable tension loops to secure the bones in place, one over the cord exiting the pisiform and the other over the group of five cords exiting the lunate. Do not trim cord ends short if the model will be dissembled and reassembled.

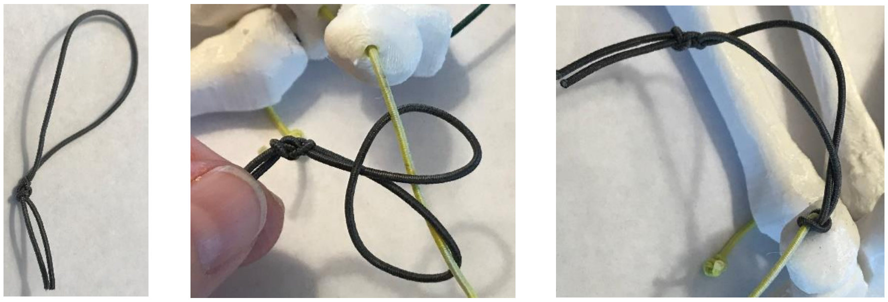

**Create tension loop and apply as shown to the cord exiting the pisiform and the cords exiting the lunate.**

 Copyright © 2018 Robin Janson 

# Learning Activities

## IV. Hand Joint Identification

1. Identify the following hand joints on the hand bone model (reference diagram on page 11):

- Distal interphalangeal (DIP)
- Proximal interphalangeal (PIP)
- Interphalangeal (IP) joint between the thumb proximal and distal phalanges
- Metacarpophalangeal (MP or MCP)
- Carpometacarpal (CMC)
- Intercarpal

## References


**Website:** American Society for Surgery of the Hand
http://www.assh.org/handcare/Anatomy/Bones#Hand

**Textbook:** Sieg, SP & Adams, KW. (2009). Illustrated Essentials of Musculoskeletal Anatomy. 5th Ed. Megabooks, Inc. ISBN-13: 978-0-935157-07-9




Copyright © 2018 Robin Janson

# Hand Bone Anatomy

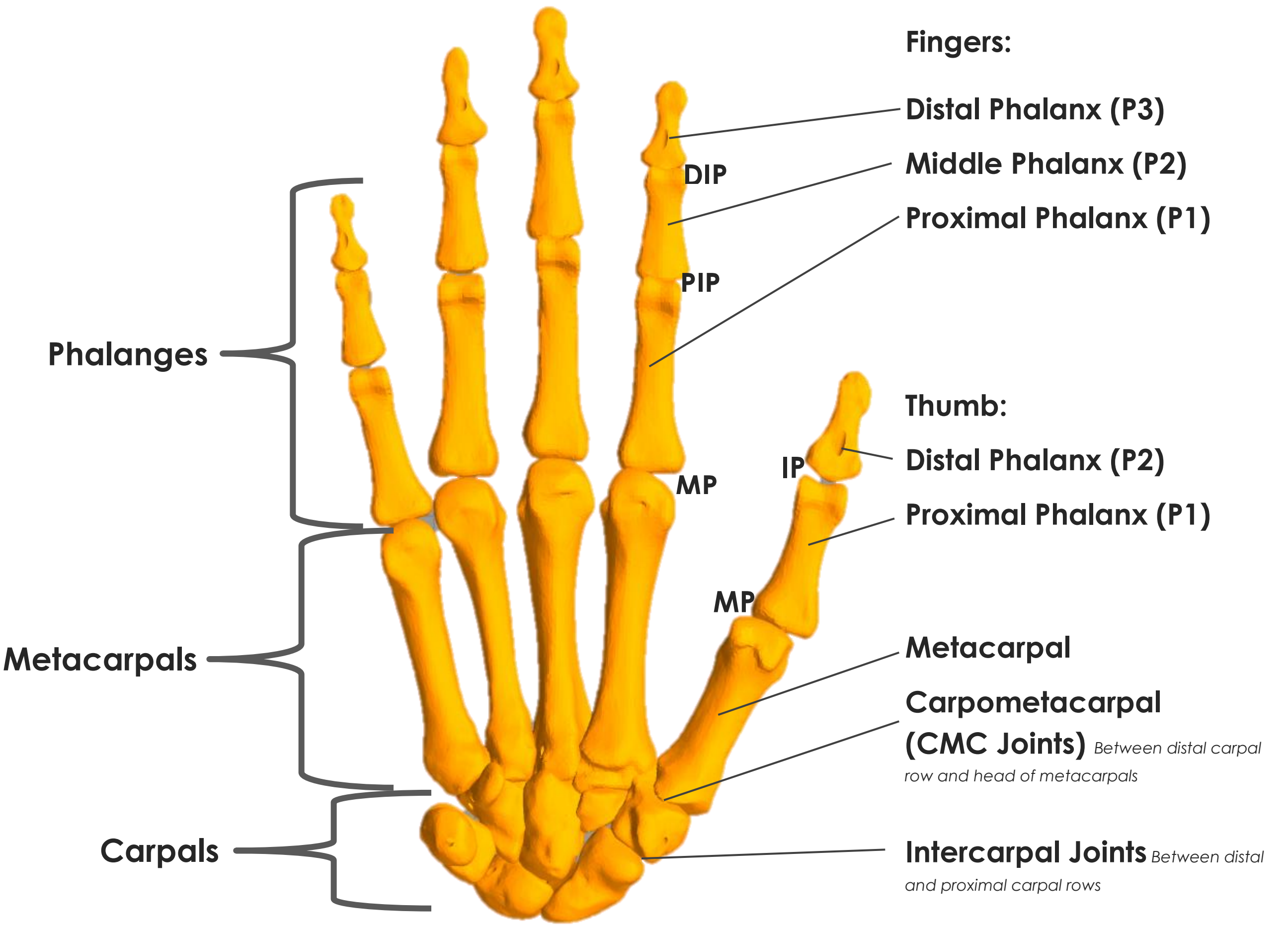


HAND RIGHT PALMAR VIEW

**Distal Carpal Row**

Trapezium[1]

Trapezoid[2]

Capitate[3]

Hamate[4]

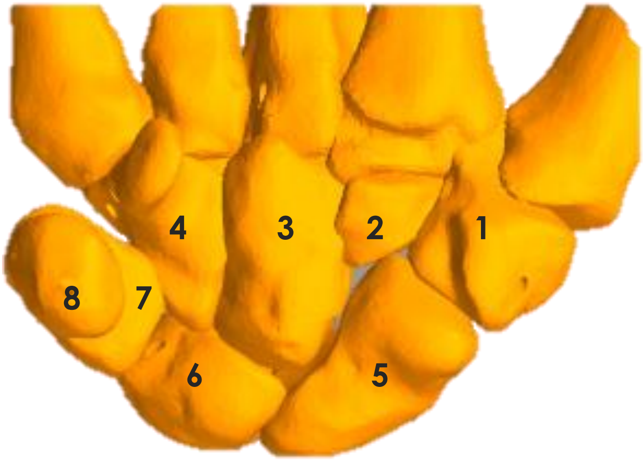


**Proximal Carpal Row**

Scaphoid[5]

Lunate[6]

Triquetrum[7]

Pisiform[8]

CARPUS RIGHT PALMAR VIEW

 Copyright © 2018 Robin Janson

# Carpal Bone Anatomy

## Trapezium
Tuberclе*
FCR Groove**

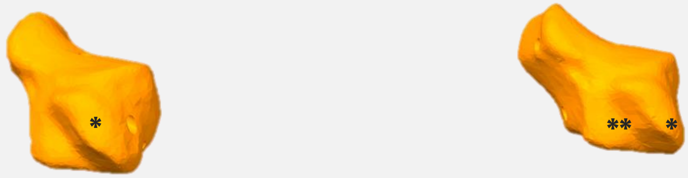


## Trapezoid

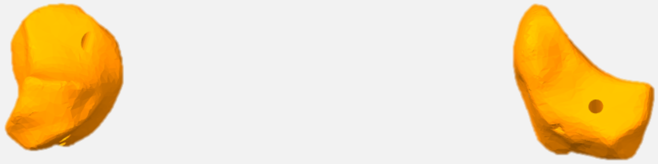

## Capitate

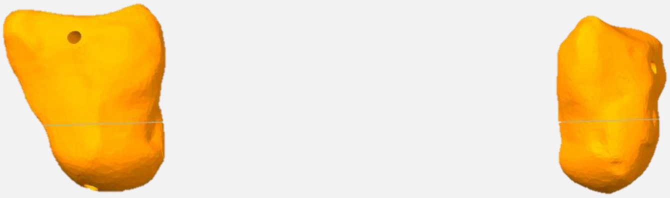

## Hamate

Hook of Hamate *

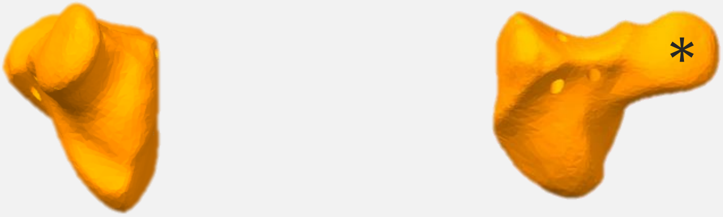


## Scaphoid
Tubercle1
Distal Pole2
Waist3
Proximal Pole4

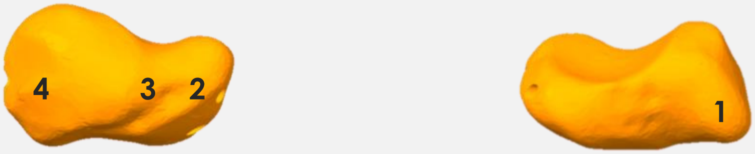


## Lunate

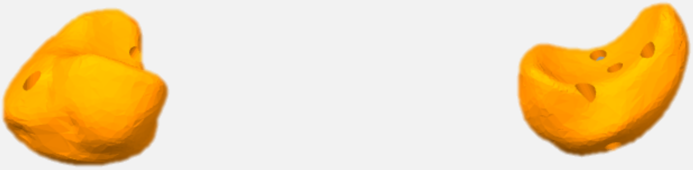

## Triquetrum

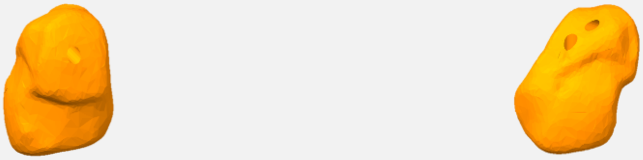

## Pisiform

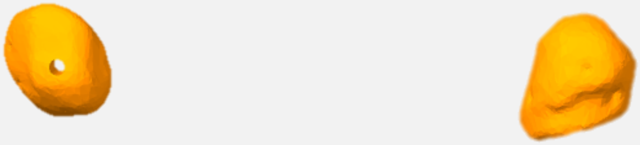

 Copyright © 2018 Robin Janson

# Hand Bone Digital Anatomy

## Thumb

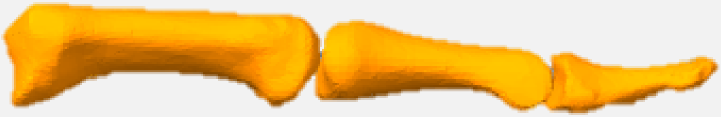


Lateral View

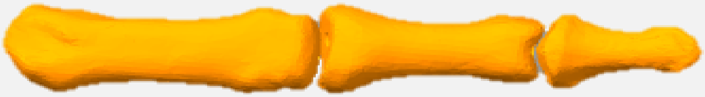

Dorsal View

## Finger (Index)

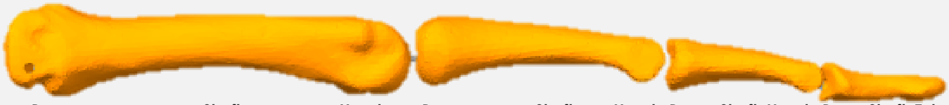


Lateral View

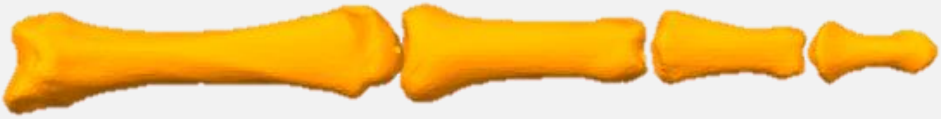

Dorsal View

## Metacarpal Bases

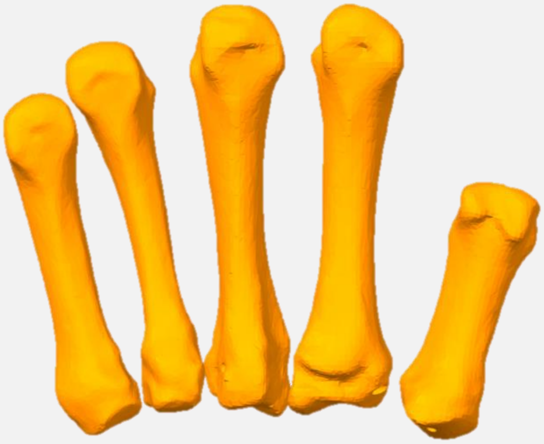

**Palm View: Small, Ring, Long, Index & Thumb**

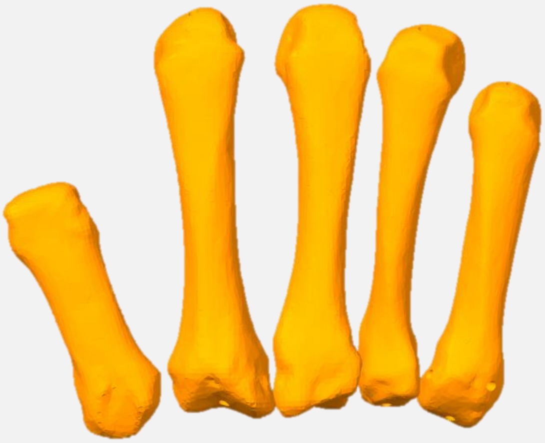

**Dorsal View: Thumb, Index, Long, Ring & Small**


Copyright © 2018 Robin Janson

# Hand Bone Anatomy Model Image

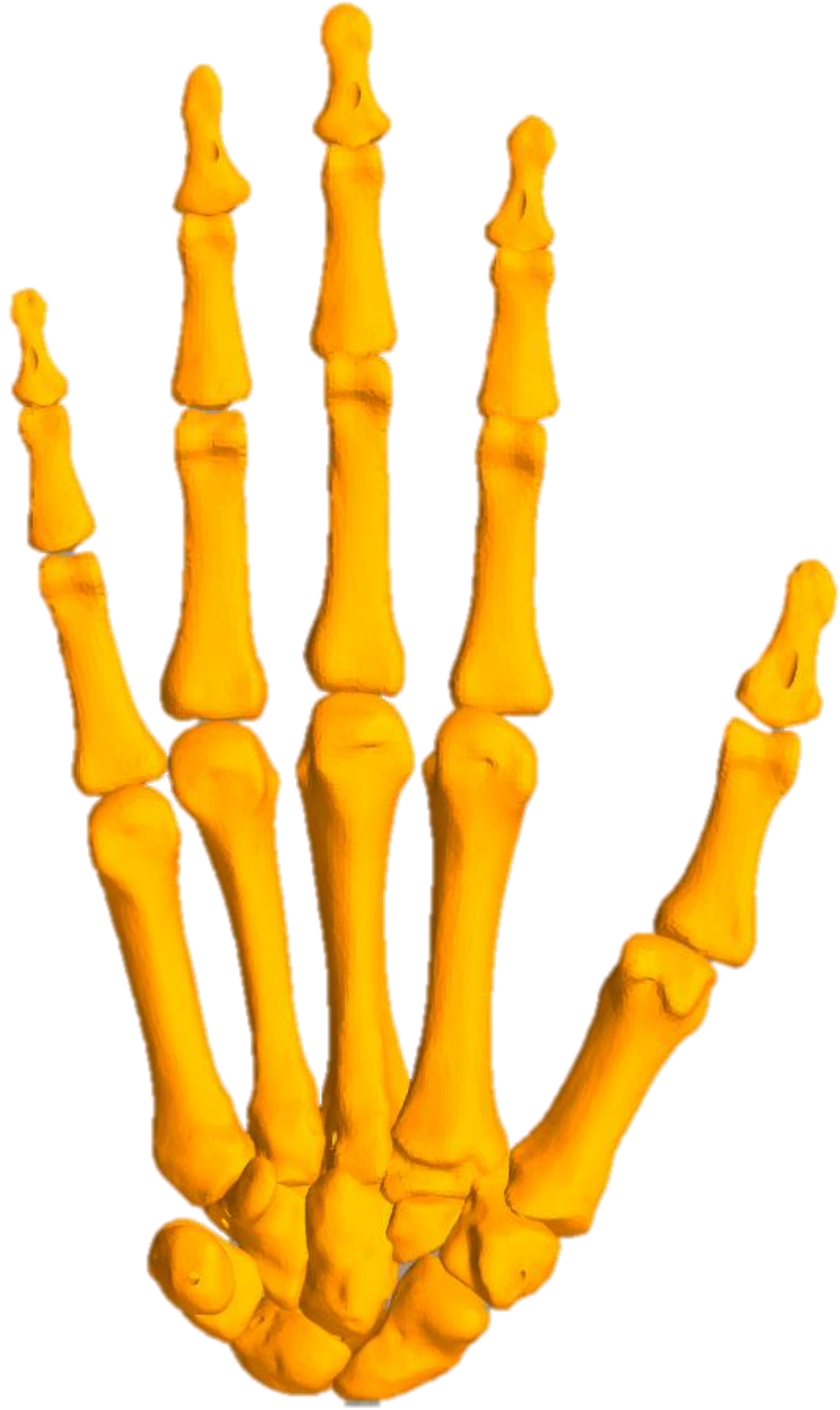

Palmar View Right Hand

 Copyright © 2018 Robin Janson

## Post-Quiz

**Instructions: After completion of the learning activities complete this post-quiz to assess your learning. Compare your pre- and post-quiz scores to assess overall gains in learning.**

1. Arrange bones in correct anatomical order (palm up/volar orientation) on a blank sheet of paper. Note that due to the shape of the wrist bones, the wrist bones will not lay in proper anatomical position.
2. Identify and label the three bone groups of the hand.
3. Identify and name each individual wrist bone.
4. Give the names of the rows of the wrist bones. List which wrist bones are in each respective row.
5. Identify and label as many special features (bony prominences) of the wrist bones as you can.
6. Identify and label the joints of the fingers, thumb and wrist.
7. Identify the three parts of the long bones of the hand?

**Answers are provided in the box below.**

**ANSWERS (Max Points = 61)**

1. Reference Hand Bone Model Diagram on page 5 for correct placement of bones. One point per correctly placed bone. Max Points= 27
2. Phalanges, metacarpals, and carpals. One point per correctly identified group. Max Points = 3
3. Scaphoid, lunate, triquetrum, pisiform, trapezium, trapezoid, capitate, hamate (pp. 5 & 12). One point per bone. Max Points=8
4. Proximal Row: scaphoid, lunate, triquetrum, pisiform / Distal Row: trapezium, trapezoid, capitate, hamate (pp. 5 & 12). One point per bone. Max Points=10 (distal row, proximal row, and the 8 bones)
5. Hamate – hook of hamate, trapezium – tubercle, scaphoid- tubercle, distal pole, waist, & proximal pole (p. 12). One point per feature. Max Points=6
6. Finger: DIP, PIP, MP & CMC; Thumb: IP, MP, CMC; intercarpal (between proximal and distal carpal rows) (p. 6). One point per joint group. Max Points=7

 Copyright © 2018 Robin Janson
